# Use of aripiprazole in an obsessive compulsive disorder case with associated motor tics

**DOI:** 10.1192/j.eurpsy.2022.1649

**Published:** 2022-09-01

**Authors:** A. Alvarado Dafonte, M. Valverde Barea, M. Solis

**Affiliations:** 1 Hospital Antequera, Usmc, Málaga, Spain; 2 Universitary Hospital of Jaén, Psychiatric, Jaén, Spain

**Keywords:** obsessive compulsive disorder, tic disorder, Aripiprazole

## Abstract

**Introduction:**

Obsessive compulsive disorder (OCD) is a pathology represented by thoughts, images, impulses or feelings that generate great anxiety and discomfort, as well as the development of compulsive acts and rituals that cause great dysfunction.

The comorbidity of different psychiatric disorders with OCD is known, such as impulse control disorder and tic disorder.

**Objectives:**

The objective of this study is to describe the clinical characteristics, comorbidities and the treatment used in a patient with an OCD diagnosis and motor tics.

**Methods:**

Description of a clinical case of motor tics associated with OCD in an adult patient.

**Results:**

A 29-year-old man begins mental health follow-up for presenting, as a result of a choking episode, obsessive thoughts with significant emotional and behavioral repercussions, to the point of restricting his diet and losing several kilos of weight. He also manifested checks and rituals in order to avoid possible choking.Treatment with sertraline and clonazepam was started, without evidence of improvement in symptoms. Months later, bucolingual and guttural tics, difficult to control by the patient and which caused bite lesions in the mouth and tongue, were added to the described clinic. Oral aripiprazole was associated to the treatment and then prolonged- release intramuscular administration was used, achieving improvement in obsessive symptoms and motor tics.

**Conclusions:**

The usefulness of adjuvant treatment with atypical antipsychotics has been demonstrated in adults with OCD who present an insufficient response to an SSRI. Injectable prolonged-release antipsychotics can help improve long-term prognosis by ensuring adherence.

**Disclosure:**

No significant relationships.

